# Neutralization of GDF15 Prevents Anorexia and Weight Loss in the Monocrotaline-Induced Cardiac Cachexia Rat Model

**DOI:** 10.3390/cells11071073

**Published:** 2022-03-23

**Authors:** Bina Albuquerque, Xian Chen, Dinesh Hirenallur-Shanthappa, Yang Zhao, John C. Stansfield, Bei B. Zhang, Abdul Sheikh, Zhidan Wu

**Affiliations:** 1 Internal Medicine Research Unit, Pfizer Worldwide Research, Development & Medical, Cambridge, MA 02139, USA; bina.albuquerque@pfizer.com (B.A.); yang.zhao6@pfizer.com (Y.Z.); beibetty.zhang@pfizer.com (B.B.Z.); abdul.sheikh@pfizer.com (A.S.); 2 Comparative Medicine, Pfizer Worldwide Research, Development & Medical, Cambridge, MA 02139, USA; xian.chen@pfizer.com (X.C.); dineshh@pfizer.com (D.H.-S.); 3 Biostatistics, Early Clinical Development, Pfizer Worldwide Research, Development & Medical, Cambridge, MA 02139, USA; john.stansfield@pfizer.com

**Keywords:** GDF15, cardiac cachexia, monocrotaline

## Abstract

Growth and differentiation factor 15 (GDF15) is a cytokine reported to cause anorexia and weight loss in animal models. Neutralization of GDF15 was efficacious in mitigating cachexia and improving survival in cachectic tumor models. Interestingly, elevated circulating GDF15 was reported in patients with pulmonary arterial hypertension and heart failure, but it is unclear whether GDF15 contributes to cachexia in these disease conditions. In this study, rats treated with monocrotaline (MCT) manifested a progressive decrease in body weight, food intake, and lean and fat mass concomitant with elevated circulating GDF15, as well as development of right-ventricular dysfunction. Cotreatment of GDF15 antibody mAb2 with MCT prevented MCT-induced anorexia and weight loss, as well as preserved lean and fat mass. These results indicate that elevated GDF15 by MCT is causal to anorexia and weight loss. GDF15 mAb2 is efficacious in mitigating MCT-induced cachexia in vivo. Furthermore, the results suggest GDF15 inhibition is a potential therapeutic approach to alleviate cardiac cachexia in patients.

## 1. Introduction

Cachexia is a multifactorial metabolic disease associated with underlying illnesses such as cancer [1], chronic heart failure (HF) [2,3], chronic kidney disease (CKD), chronic obstructive pulmonary disease (COPD), chronic inflammation, and sepsis [1,2,3,4]. Cardiac cachexia is prevalent in patients with congestive heart failure (CHF) and is associated with poor prognosis [2,3,5,6,7]. Anorexia (loss of appetite), unintentional weight loss, muscle wasting, fat loss, and fatigue are the hallmark symptoms of cardiac cachexia [4,8] and negatively impact the quality of life and survival in patients [2,4]. Currently, there is no approved therapy for this debilitating condition. Thus, new therapy is urgently needed for cardiac cachexia.

Growth and differentiation factor 15 (GDF15) is a cytokine of the transforming growth factor β family reported to cause anorexia, emesis, and weight loss in rodents, musk shrews, and nonhuman primates [9,10,11,12,13,14,15,16,17,18]. GDF15 regulates energy metabolism by activating its receptor, glial cell-derived neurotrophic factor receptor alpha-like (GFRAL), expressed selectively in the area postrema and nucleus of the solitary tract of the hindbrain [10,11,14,15,19]. Elevated circulating GDF15 is associated with cachexia and poor survival in cancer patients [20,21]. We found that GDF15 was higher in cancer patients receiving platinum-based chemotherapy and is positively associated with weight loss [9], supporting its role in mediating platinum-induced side-effects of emesis, nausea, and weight loss. Blocking the GDF15/GFRAL pathway genetically or pharmacologically has demonstrated robust effects in mitigating anorexia and weight loss induced by tumor and/or cisplatin in preclinical models [9,11,22,23].

Interestingly, GDF15 levels have been reported to be elevated in patients with HF and negatively associated with clinical outcome and mortality [24,25,26,27]. Higher levels of GDF15 were found in patients with HF who were older and with higher New York Heart Association functional class and had lower body mass index and exercise capacity [27]. However, it is unknown if GDF15 is causal in the development of cardiac cachexia and if inhibition of the GDF15 pathway could be beneficial in this disease setting. Here, we examined the relationship of GDF15 with the development of cachexia in the monocrotaline (MCT)-induced cardiac cachexia rat model [28]. Furthermore, we investigated the causal role of GDF15 by assessing whether GDF15 blockade via a selective monoclonal antibody (mAb2) could prevent MCT-induced anorexia, weight loss, fat loss, and muscle atrophy in this preclinical cardiac cachexia model.

## 2. Materials and Methods

### 2.1. Materials

Anti-GDF15 mAb2 and immunoglobulin G (IgG) isotype control were generated at Pfizer. GDF15 mAb2 is a humanized monoclonal antibody that binds GDF15 and blocks its interaction with GFRAL. It was characterized previously [29]. MCT was purchased from Oakwood Chemical (Cat.no. 002602-1g; Estill, SC, USA).

### 2.2. Methods

#### 2.2.1. In Vivo Rat Study

Sprague-Dawley male rats purchased from Charles River Laboratories (Wilmington, MA, USA), aged 7–8 weeks (<300 g body weight), housed in standard room temperature (22 ± 1 °C) conditions, were used for the study. Rats were injected intraperitoneally with MCT at 50 mg/kg on day 1 (single injection administered in the morning) and subcutaneously with GDF15 mAb2 or control IgG once every 3 days from day 1 through the duration of the study. Data shown here were conducted in two separate studies—a validation study comprising vehicle- and MCT-treated animals that was 24 days in duration, and a GDF15 mAb2 study comprising vehicle (non-MCT–IgG-treated), MCT–IgG (MCT + IgG-treated), and MCT–mAb2 (MCT + mAb2 treated) that was 18 days in duration. All procedures performed on animals were approved by the Pfizer Groton and Cambridge Animal Care and Use Committee.

#### 2.2.2. Body Weight, Food Intake, and Body Composition Assessment

Body weight and food intake were measured at approximately 08:00–10:00 a.m. using a digital scale (Mettler Toledo, Columbus, OH, USA). Body composition was measured via EchoMRI^®^ (4-in-1, Houston, TX, USA) at the timepoints indicated.

#### 2.2.3. Echocardiography

Echocardiography was performed to assess left-ventricular (LV) and right-ventricular (RV) function at ~2–3 and 4 weeks post MCT injection. Echocardiography imaging in rat was performed similar to a previously published procedure in mouse and rat [30,31]. Briefly, rats were anesthetized using isoflurane (3%) in an induction chamber, and then anesthetic status was maintained at ~2% isoflurane during animal preparation and image acquisition. While anesthetized, an animal was transferred onto a water-circulating heated blanket to remove hair around the left lateral and ventral thoracic area using a chemical hair removal agent. After hair removal, each animal was transferred onto a heated (~34–35 °C) platform for echocardiography.

Transthoracic parasternal long axis B-mode and parasternal short axis M-mode images of the heart, as well as pulse wave doppler images of the pulmonary artery, were acquired using the 18–38 MHz transducer (VisualSonics MS400^®^) in a Vevo 2100^®^ ultrasound (Fujifilm Visualsonics Corporation, Bothell, WA, USA) machine to assess LV and RV structure and function. Anesthesia level was adjusted to maintain heart rate ~350–450 bpm during image acquisition. After image acquisition, the animal was transferred to a warm cage for recovery. Post image acquisition, images were analyzed using Vevo lab^®^ analysis software, and the following parameters were collected from image analysis: LV ejection fraction, LV dimensions, pulmonary artery peak flow rate, pulmonary artery flow acceleration time, right-ventricle area, and heart rate.

#### 2.2.4. Plasma Marker Measurement

Rat plasma (not fasted) was collected at termination from the validation study. Rat plasma GDF15 was measured using ELISA (R&D Systems, Minneapolis, MN, USA). Plasma cytokines were measured using a Meso-scale Discovery kit (V-PLEX Proinflammatory Panel 1 Human Kit, cat.no. K15049D-1).

#### 2.2.5. Measurement of Muscle Gene Expression by Quantitative Polymerase Chain Reaction (qPCR)

Total RNA was extracted and purified using QIAzol Lysis Reagent (Qiagen, Cat.: 79306, Germantown, MD, USA) from rat tibialis anterior muscle and then reverse-transcribed into cDNA with a High-Capacity cDNA Reverse Transcription Kit (Cat.: 4374966; Applied Biosystems Inc., Foster City, CA, USA). Catabolism genes in skeletal muscle (Atrogin-1 (Fbxo32); muscle ring finger 1, MuRF1 (Trim63); Foxo1 (forkhead box O1)) were examined by qPCR and normalized to the control gene TATA-binding protein (TBP).

#### 2.2.6. Statistical Analysis

**Figure 1:**
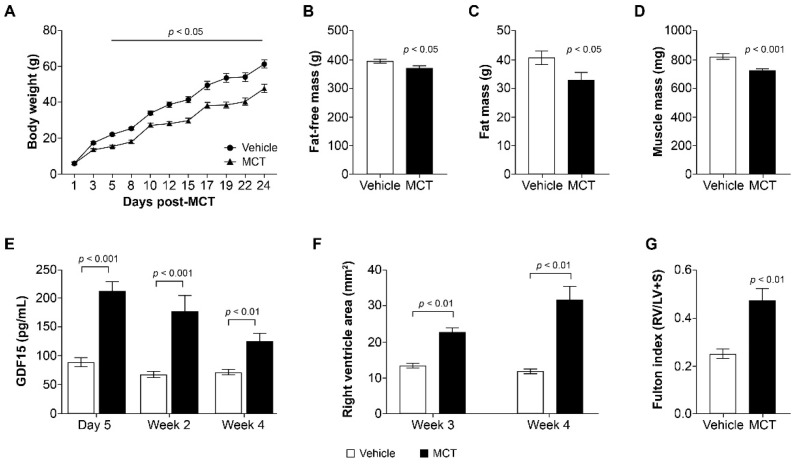
MCT treatment increased GDF15 and caused cachexia and RVD in rats. (**A**) Change in body weight (g) from baseline on days 1–24; *p* < 0.05 MCT group vs. vehicle from day 5 onward. Data were analyzed with a longitudinal mixed-effects model with an AR(1) covariance structure. (**B**) Fat-free mass at 3 weeks post MCT; *p* < 0.05 MCT group vs. vehicle. Data were analyzed using a Welch’s two-sample *t*-test. (**C**) Fat mass at 3 weeks post MCT; *p* < 0.05 MCT group vs. vehicle. Data were analyzed using an unpaired *t*-test. (**D**) Muscle mass (tibialis anterior) at 3 weeks post MCT; *p* < 0.001 MCT group vs. vehicle. Data were analyzed using a Welch’s two-sample *t*-test. (**E**) Circulating GDF15 levels (pg/mL) measured at day 5, week 2, and week 4; *p* < 0.01 week 4, *p* < 0.001 day 5 and week 2 MCT group vs. vehicle. Data were analyzed with a longitudinal mixed-effects model with an AR(1) covariance structure. (**F**) Right-ventricular dysfunction according to right-ventricular area (mm^2^) measured at weeks 3 and 4; *p* < 0.01 MCT group vs. vehicle. Data were analyzed with a longitudinal mixed-effects model with an AR(1) covariance structure. (**G**) Fulton index (RV/LV + S) measured at week 4; *p* < 0.01 MCT group vs. vehicle. Data were analyzed using a Welch’s two-sample *t*-test. Data are presented as the least squares mean ± SEM. ANOVA: analysis of variance; GDF15: growth and differentiation factor 15; LV: left ventricle; MCT: monocrotaline; RV: right ventricle; RVD: right-ventricular dysfunction; SEM: standard error of the mean; S: septum.

**Figure 2:**
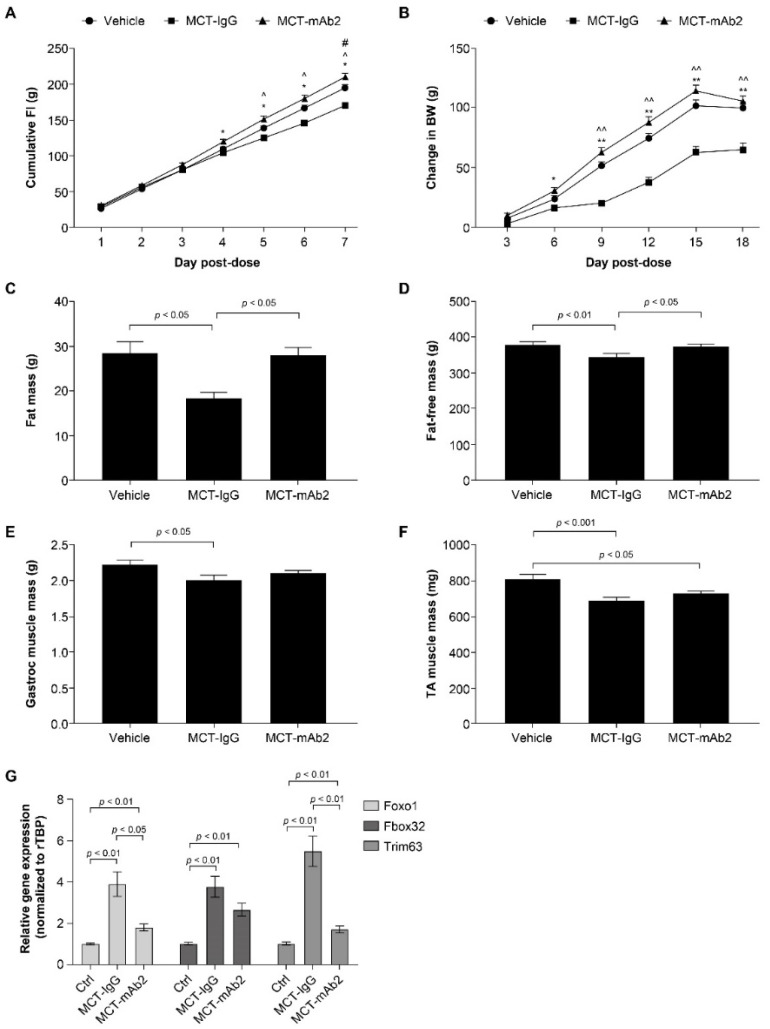
GDF15 mAb2 treatment prevented MCT-induced anorexia and weight loss. (**A**) Cumulative food intake at days 1–7 post MCT and GDF15 mAb2 dosing. * *p* < 0.05 MCT–IgG vs. MCT–mAb2, ^ *p* < 0.05 MCT–IgG vs. vehicle, # *p* < 0.05 MCT–mAb2 vs. vehicle. Data were analyzed with a longitudinal mixed-effects model with an AR(1) covariance structure. (**B**) BW change at days 3–18 post MCT and GDF15 mAb2 dosing; * *p* < 0.05, ** *p* < 0.01, MCT–IgG vs. MCT–mAb2, ^^ *p* < 0.01, MCT–IgG vs. vehicle. Data were analyzed with a longitudinal mixed-effects model with an AR(1) covariance structure. (**C**) Fat mass at day 17 post MCT. Data were analyzed using one-way ANOVA and a Tukey HSD test. (**D**) Fat-free mass at day 17 post MCT. Data were analyzed using one-way ANOVA and a Tukey HSD test. (**E**) Muscle mass (gastrocnemius) at 3 weeks post MCT. Data were analyzed using one-way ANOVA and a Tukey HSD test. (**F**) Muscle mass (tibialis anterior) at 3 weeks post MCT. Data were analyzed using one-way ANOVA and a Tukey HSD test. (**G**) mRNA expression of Foxo1, Fbox32, and Trim63 in tibialis anterior muscle. Data were analyzed using pairwise Wilcoxon tests. Data are presented as the least squares mean ± SEM. ANOVA: analysis of variance; BW: body weight; Ctrl: control; FI: food intake; Gastroc: gastrocnemius; GDF15: growth and differentiation factor 15; IgG: immunoglobulin G; mAb: monoclonal antibody; MCT: monocrotaline; SEM: standard error of the mean; TA tibialis anterior; rTBP: rat TATA-binding protein.

**Figure 3:**
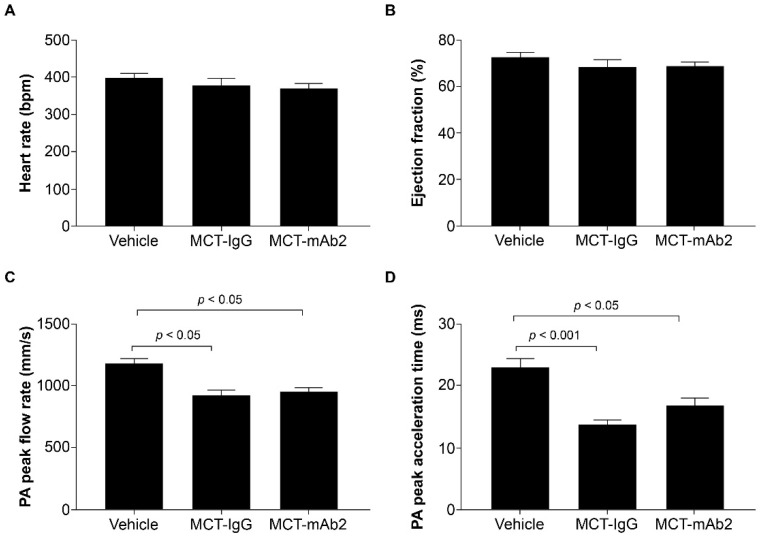
No effect of GDF15 mAb2 treatment on MCT-induced cardiac function impairment. (**A**) Heart rate (bpm) at day 16 post-MCT and GDF15 mAb2 dosing. Data were analyzed using one-way ANOVA. (**B**) Ejection fraction (%) at day 16 post-MCT and GDF15 mAb2 dosing. Data were analyzed using one-way ANOVA. (**C**) PA peak flow rate (mm/s) at day 16 post-MCT and GDF15 mAb2 dosing. Data were analyzed using one-way ANOVA and Tukey HSD test. (**D**) PA peak acceleration time (ms) at day 16 post-MCT and GDF15 mAb2 dosing. Data were analyzed using one-way ANOVA and Tukey HSD test. Data represented as least squares mean ± SEM. ANOVA: analysis of variance; GDF15: growth and differentiation factor 15; IgG: immunoglobulin G; mAb: monoclonal antibody; MCT: monocrotaline; PA: pulmonary arterial; SEM: standard error of mean.

Data are expressed as means ± SEM. Longitudinal mixed-effects models with fixed effects for treatment group and time, random effects for animal ID, and AR(1) covariance structures were fit to compare the longitudinal measurements of body weight, GDF15 levels, RV area, and cumulative food intake. Welch’s two-sample *t*-tests were used to compare fat mass, fat-free mass, muscle weight, and Fulton index measurements between MCT and vehicle groups (Figure 1). We used pairwise Wilcoxon tests to compare between the groups for the mRNA expression data (Figure 2G). One way analysis of variance and Tukey HSD post hoc tests were used for comparing all other measurements at a single timepoint across control, MCT vehicle, and MCT mAb2 treatment groups (Figure 2 and Figure 3). All statistical analyses were performed in R 4.0.5.

## 3. Results

### 3.1. MCT Increased Circulating GDF15 and Reduced Body Weight in Rats

GDF15 was reported to be elevated in MCT-treated rats, a cardiac cachexia model [28,32], suggesting it might play a role in the development of cachexia induced by MCT. To investigate if GDF15 is causal in driving cachexia in this model, we first determined the effect of MCT on circulating GDF15 and body weight. MCT dosing resulted in a significant decrease in body weight starting at day 5 post dose through the duration of the study (Figure 1A). Fat-free mass and fat mass were also significantly reduced compared with vehicle controls at 3 weeks post dose (Figure 1B,C). Tibialis anterior muscle mass measured at necropsy was also significantly lower in the MCT-treated group versus vehicle control (Figure 1D). Circulating GDF15 levels were measured at day 5, week 2, and week 4 post MCT dose. Plasma GDF15 levels were elevated more than twofold at day 5 post MCT treatment. The circulating level in the MCT-treated group remained approximately twofold of that in the vehicle control at weeks 2 and 4 (Figure 1E). Other cytokines (INF-γ, IL-1β, IL-4, IL-6, IL-10, IL-13, TNF-α) were also increased by MCT treatment (data not shown). Thus, the MCT rat model presents with anorexia-associated body weight loss with increased circulating GDF15 and cytokine levels after MCT dosing. Consistent with previous reports, MCT administration resulted in right-ventricular dysfunction (RVD) in our study. At 3 and 4 weeks, increased right-ventricle area and Fulton index (measured as right-ventricle weight/left-ventricle weight + septum) were observed in the MCT-treated group compared with the vehicle control group (Figure 1F,G).

### 3.2. GDF15 mAb2 Treatment Prevents MCT-Induced Anorexia and Weight Loss

The positive association of circulating GDF15 with weight loss in rats prompted us to examine if elevated GDF15 by MCT is causal to cachexia in vivo. Rats were cotreated with MCT and either an anti-GDF15 monoclonal antibody (mAb2) or control IgG for a duration of 2.5 weeks. Cumulative food intake at day 1 to 7 post MCT and GDF15 mAb2 dosing is shown in Figure 2A. MCT dosing significantly reduced cumulative food intake starting from day 5 onward. Cotreatment with GDF15 mAb2 completely prevented MCT-induced reduction of food intake (Figure 2A). Concomitant with the reduction in food intake, body weight gain in the MCT-treated group was significantly less than that observed in the vehicle-treated group (Figure 2B). Cotreatment with MCT and GDF15 mAb2 restored the weight gain comparable to the vehicle group starting at day 9 through the duration of the study (Figure 2B). MCT administration resulted in a 40% reduction in fat mass (Figure 2C) and 10% reduction in fat-free mass (Figure 2D) when compared with the vehicle-treated group. Fat mass and fat-free mass reductions were completely mitigated with mAb2 dosing (Figure 2C,D). MCT significantly reduced muscle mass (tibialis anterior and gastrocnemius) relative to the vehicle group (Figure 2E,F). Reductions in gastrocnemius and tibialis anterior muscle mass induced by MCT were attenuated by mAb2 treatment but did not reach statistical significance (Figure 2E,F). Increased protein breakdown by activation of catabolism genes in muscle has been implicated in muscle wasting in cachexia [33,34]. MCT was reported to induce the expression of Trim63 (MurF1) and Fbox32 (Atrogin-1), two E3 ligases involved in protein ubiquitination and degradation in muscle [35,36]. We measured the expression of Trim63 (MurF1) and Fbox32, as well as their upstream regulator Foxo1 in tibialis anterior muscle. The mRNA level of all three genes was markedly increased by MCT in the MCT–IgG group compared with the vehicle group (Figure 2G). Treatment with GDF15 mAb2 significantly attenuated the elevation of gene expression by MCT (Figure 2G), suggesting that GDF15 inhibition counteracts the perturbation of catabolism gene expression in muscle.

### 3.3. GDF15 mAb2 Treatment Does Not Improve MCT-Induced Cardiac Dysfunction in Rats

To determine if the beneficial effects of GDF15 neutralization observed in MCT-treated rats resulted from improvement of pulmonary and cardiac function, we assessed heart rate, ejection fraction, pulmonary arterial (PA) flow acceleration time, and PA peak flow rate at day 16 post MCT and mAb2 treatment. There were no differences in heart rate and ejection fraction observed across all groups (Figure 3A,B). MCT reduced PA peak flow rate and PA peak acceleration time significantly in the MCT–IgG group compared with the vehicle group (Figure 3C,D). However, rats treated with MCT and mAb2 also showed significantly lower PA peak flow rate and peak acceleration time compared with the vehicle group. PA peak flow rate and acceleration were comparable between MCT–mAb2 and MCT–IgG groups indicating that mAb2 treatment does not improve MCT-induced RVD. Thus, the effects of GDF15 mAb2 on mitigating MCT-induced food intake and weight loss are not due to pulmonary and cardiac function improvement.

## 4. Discussion

Cardiac cachexia is a debilitating condition in patients with CHF and is associated with worse clinical outcomes and poor survival [2,3,5,6,7]. Anorexia, elevation of proinflammatory cytokines, and imbalanced protein degradation/synthesis in skeletal muscle have been implicated in weight loss and muscle wasting [2,37,38]; however, there is currently no approved therapy available to target the underlying pathogenesis and improve cardiac cachexia. GDF15 has been reported to play a causal role in cancer cachexia, and GDF15 neutralization appears to be efficacious in mitigating tumor-induced cachexia preclinically [9,11,20,21,22,23]. GDF15 was also found to be elevated in CHF and PAH patients [24,32,39]; however, it is unknown if GDF15 is causal to the development of cachexia in these patients. To examine if GDF15 plays a role in cardiac cachexia preclinically, we determined the effect of GDF15 neutralization on the development of cachexia in MCT-induced PAH and cardiac cachexia rat model.

MCT is a pneumotoxic agent commonly used to induce PAH by remodeling of the pulmonary vasculature that leads to RVD and ultimately HF in rodents [28,32,36,40,41]. Cachexia and elevated GDF15 levels were also observed in MCT-treated rats [8,42,43,44]. We used this cardiac cachexia rat model to investigate the role of GDF15 in MCT-induced cachexia. We first characterized the effect of MCT on GDF15 circulating levels, weight loss, and body composition in rats. Consistent with previous reports [45,46], MCT-induced RVD was indicated by increased RV area and the Fulton index, as expected. MCT increased circulating GDF15 levels at least twofold throughout the duration of the study. Concomitant with elevation of GDF15, the rats dosed with MCT developed cachexia, demonstrated by reduced food intake and loss of body weight, fat, and lean mass, as well as muscle atrophy. To determine if elevated GDF15 levels by MCT are causal for the development of cachexia, rats were cotreated with a selective and potent GDF15 antibody (mAb2) with MCT to block GDF15 activity. GDF15 mAb2 completely prevented MCT-induced anorexia and weight loss, including lean and fat mass loss. GDF15 mAb2 treatment showed a trend in attenuating the reduction in tibialis anterior and gastrocnemius muscle mass in rats dosed with MCT but this effect did not reach statistical significance. Interestingly, we observed a robust increase in Foxo1, Trim63, and Fbox32 in tibialis anterior muscle from MCT-treated mice, suggesting that there is an increase in protein ubiquitination and degradation. This increase was significantly attenuated by GDF15 mAb2 treatment. A longer duration of treatment might be needed to improve muscle atrophy. The data demonstrate that GDF15 is essential in mediating MCT-induced anorexia and weight loss in vivo and is causal to the development of cachexia in this model. GDF15 mAb2 treatment does not prevent MCT-induced cardiac dysfunction, indicating that the efficacy on cachexia prevention is independent of cardiac function in this model. Additional therapy may be required to have direct effects on improving cardiac function and potentially an effective combination with GDF15 neutralization.

Our results, for the first time, demonstrate that elevated circulating GDF15 is causal to anorexia and weight loss in a cardiac cachexia preclinical model. In addition, GDF15 neutralization has robust efficacy in mitigating cachexia in this model. Although GDF15 mAb2 treatment did not improve cardiac function in the MCT model, it remains to be determined if this finding is specific to the MCT model. Future studies to test the efficacy of GDF15 mAb2 in additional cardiac cachexia models will increase our confidence in the translatability of our findings. Our results provide mechanistic insights into the underlying pathogenesis contributing to cardiac cachexia and new therapeutic approaches to treat this disease. Future studies will be needed to determine if GDF15 neutralization is efficacious in reversing MCT-induced cachexia and improving physical performance and survival. Our data suggest that GDF15 plays a role in cardiac cachexia, and GDF15 neutralization could potentially be an effective therapeutic approach for the treatment of cachexia in HF or other chronic diseases with elevated GDF15, such as COPD, CKD, and cancer.

## Data Availability

The data presented in this study are available on request from the corresponding author. The data are not publicly available.
